# Platelets in Immune Response to Virus and Immunopathology of Viral Infections

**DOI:** 10.3389/fmed.2018.00121

**Published:** 2018-04-30

**Authors:** Eugenio D. Hottz, Fernando A. Bozza, Patrícia T. Bozza

**Affiliations:** ^1^Laboratório de Imunofarmacologia, Instituto Oswaldo Cruz, Fundação Oswaldo Cruz, Rio de Janeiro, Brazil; ^2^Departamento de Bioquimica, Universidade Federal de Juiz de Fora, Juiz de Fora, Brazil; ^3^Laboratório de Medicina Intensiva, Instituto Nacional de Infectologia Evandro Chagas, Fundação Oswaldo Cruz, Rio de Janeiro, Brazil; ^4^Instituto D’Or de Pesquisa e Ensino, Rio de Janeiro, Brazil

**Keywords:** platelet, HIV infection, dengue, influenza, immunology and virology, viruses

## Abstract

Platelets are essential effector cells in hemostasis. Aside from their role in coagulation, platelets are now recognized as major inflammatory cells with key roles in the innate and adaptive arms of the immune system. Activated platelets have key thromboinflammatory functions linking coagulation to immune responses in various infections, including in response to virus. Recent studies have revealed that platelets exhibit several pattern recognition receptors (PRR) including those from the toll-like receptor, NOD-like receptor, and C-type lectin receptor family and are first-line sentinels in detecting and responding to pathogens in the vasculature. Here, we review the main mechanisms of platelets interaction with viruses, including their ability to sustain viral infection and replication, their expression of specialized PRR, and activation of thromboinflammatory responses against viruses. Finally, we discuss the role of platelet-derived mediators and platelet interaction with vascular and immune cells in protective and pathophysiologic responses to dengue, influenza, and human immunodeficiency virus 1 infections.

## Introduction

Platelets are highly specialized effectors of hemostasis with essential functions in vascular thrombosis and wound healing. For a long time, cellular activities attributed to platelets were restricted to rapid procoagulant responses mediated by G-protein-coupled receptors leading to platelet aggregation, enzymatic activation of eicosanoid synthesis and granule secretion (1, 2). Aside from this traditional view, it is now known that platelets express various pattern recognition receptors (PRR) and respond to infecting microorganisms, initiating cellular activities that participate in the immune and inflammatory network against diverse pathogens, including viruses (1–3). While traditional platelet activation by G-protein-coupled receptors is usually rapid, platelet PRR activation in responses to infectious and immune stimuli can be delayed and sustained, lasting hours after initial aggregation and secretion (1, 4). For example, platelets have stored RNA molecules and diverse mechanisms for posttranscriptionaly process intronic RNA using specialized pathways to synthesize immunoregulatory proteins such as cytokines and antimicrobial peptides (5–7). Through this new view, platelets are now recognized as first-line sentinels in detecting and responding to pathogens and damage signals in the vasculature and also in the extravascular space.

Pattern recognition receptors are cellular sensors that recognize molecular structures broadly shared among pathogens, the so called pathogen-associated molecular patterns (PAMPs); or cellular molecules modified and/or released during tissue damage called damage-associated molecular patterns (DAMPs). PRR from different classes have been shown to be expressed and functional in platelets, including those from C-type lectin receptors (CLR) and toll-like receptors (TLRs) families (2, 8, 9). CLR are a large family of surface proteins containing at least one carbohydrate-binding domain which are specialized in the recognition of bacterial, fungi, or viral glycans (10–12). Some viruses can exploit certain CLR for viral attachment and entry in host cells, including in platelets (13, 14). The TLRs, a family of transmembrane cellular sensors, are the best described class of PRR in platelets. While virtually all TLRs (1–10 in human) are detected at some level (mRNA or protein) in platelets (1–3, 8, 15–18), most of functional characterization of TLR-mediated platelet responses were reported for those specialized in bacterial molecules, especially regarding platelet TLR-4 activation by lipopolysaccharide (LPS) from Gram-negative bacteria (4, 6, 19–22). Recently, the expression and functionality of endosomal TLR-3, -7, and -9, related to recognition and response to viral genome in nucleated cells, were also reported in platelets (23–25). The presence of cytoplasmic PRR in platelets is far less explored, and it remains a subject of controversy whether platelets are able to sustain the replication of viral genome allowing its recognition by cytoplasmic sensors.

Two major cytoplasmic PRR from the NOD-like receptor (NLR) family were recently reported to be expressed and functional in platelets: the nucleotide-binding domain leucine rich repeat containing pyrin 3 (NLRP3), a major sensor for the activation of inflammasome that recognizes various bacterial, viral, and tissue damage signals; and the nucleotide-binding oligomerization domain 2 that recognizes the bacterial cell wall peptidoglycan component muramyl dipeptide (26–28). Other intracellular sensors including retinoic acid-inducible gene I and melanoma differentiation-associated gene 5, which are highly specialized in viral RNA recognition, are expressed in human megakaryocytes in response to type I interferon (IFN-α and -β) (29), but their expression in platelets, as well the ability of platelets to express other IFN-stimulated genes (ISGs) and to perform IFN-induced antiviral response are not known.

Megakaryocytes, platelets’ mother cells, transfer to platelets all cellular components responsible for their hemostatic and immune functions including granule-stored chemokines, immune receptors, RNA molecules, and spliceosomes (5, 23, 30, 31). Megakaryocytes have been shown to be susceptible to various viruses (29, 32–37). In addition, megakaryocytes express PRR and cytokine receptors, and there is evidence that TLR agonists or cytokine engagement affects megakaryocytic maturation and thrombopoiesis (25, 34, 38, 39). Megakaryocytes and megakaryocytic cell lines respond to viral infections or viral PAMPs by secreting high levels of α and β IFN (25, 29, 34, 40, 41), which reduce platelet production *in vitro* through an autocrine IFNAR signaling (25, 40–42). Besides their consequences reducing the numbers of new platelets, megakaryocyte infection, PRR engagement, and/or cytokine signaling are expected to change the phenotype of platelet progeny during infections, influencing platelet-mediated immune and inflammatory processes at the periphery. However, participation of megakaryocytes in immune response still deserves more in-depth investigation. In this review, we discuss the molecular mechanisms involved in platelet interactions with viruses including PRR and intracellular pathways related to platelet inflammatory activities during viral infections. We focused our discussion on the contributions of human platelets to pathophysiologic and protective responses during viral infections of major concern in human health globally including acquired immunodeficiency syndrome (AIDS), dengue hemorrhagic fever/dengue shock syndrome (DHF/DSS), and influenza pneumonia.

## Platelet Interaction and Response to Virus

**Figure 1:**
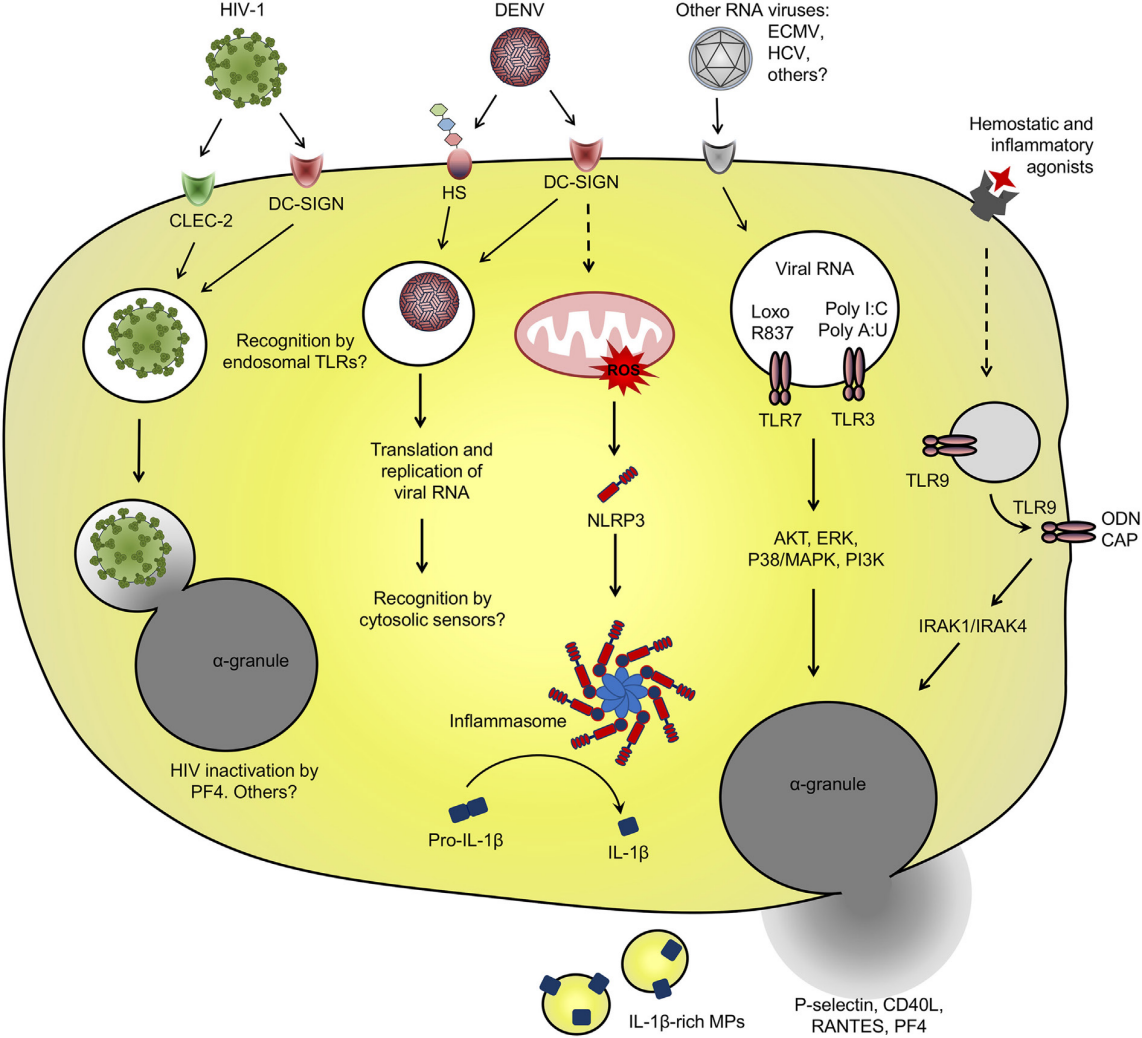
Platelet interaction with viruses and virus-related pathogen-associated molecular patterns (PAMPs): schematic representation of the main receptors and pathways involved in virus binding and internalization, and pattern recognition receptor involved in recognition of viral PAMP by platelets. See text for details and references.

Viral infection of a susceptible cell initiates with virus binding to a surface receptor that mediate its internalization through the endocytic pathway. Successful infection relies on the ability of the virus to escape endosomal acidification and lysosomal fusion through diverse mechanisms, delivering its genome to the cytoplasm or nucleus depending on the virus type (43–45). For each step of viral replication, the host cell has evolved mechanisms for recognizing and fighting viral infection, mainly by inducing the expression of antiviral restriction factors in bystander cells through type I IFN signaling (46–48). Platelets have been reported to express surface receptors able to mediate binding and entry of various viruses [reviewed in Ref. (49–51)]. Even though platelets do not have nucleus, they have all the molecular machinery to synthesize proteins from stored mRNA (5, 17, 52, 53), which may also implicate in the ability to translate proteins from RNA viruses. In addition, some of the PRR related to viral recognition were recently identified to be present and functional in platelets, and platelet participation in immune response to virus has been investigated in experimental viral infections and in human patients naturally infected with viruses (23–26, 54–56). Recent studies of these types have increased our understanding of how platelet responses triggered by interactions with viruses may both limit viral proliferation and pathogen burden, or complicate inflammation and disease pathogenesis. The main mechanisms for platelet interaction with viruses and the PRR involved in viral recognition by platelets known so far are discussed in this chapter and summarized in Figure 1.

Among diverse surface receptors involved in platelet interactions with viruses (49–51), the CLR dendritic cell-specific ICAM-3-grabbing non-integrin (DC-SIGN) is involved in dengue virus (DENV) and human immunodeficiency virus 1 (HIV-1) binding and entry in platelets (57, 58). DC-SIGN is a PRR that recognizes mannose-terminal-containing pathogen-associated carbohydrates. DC-SIGN also binds glycosylated domains on the envelope (E) protein of DENV and glycoprotein 120 (GP120) of HIV-1, a recognized mechanism for viral entry in dendritic cells (13, 45, 50, 59, 60). HIV-1 entry in dendritic cells through DC-SIGN or other C-type lectins occurs without viral membrane fusion and independently on the receptor CD4 or the coreceptors CXCR4 or CCR5 (13, 45, 50). Through this non-canonical pathway, endocytosed HIV particles sustain their infectivity for several days allowing trans-infection to CD4^+^ T cells (13, 45). Even through platelets internalize HIV-1 viral particles though similar mechanisms involving DC-SIGN or C-type lectin-like receptor 2 (CLEC-2) for viral attachment (14, 57), it is still controversial if platelets can perform HIV-1 trans-infection to T cells, and opposite results have been found on this regard (14, 61), as will be discussed in Section “Platelet Activation in HIV Infection.”

Platelet ability to endocytose viral particles is not an HIV-specific feature. The viral genome of DENV and hepatitis C virus (HCV), both from Flaviviridae family, have been detected in circulating platelets from infected patients (62–67). HCV genome was also detected in platelets from healthy volunteers that were exposed to HCV *in vitro* (65, 68). HCV capture by platelets depended on the binding domain of HCV envelope protein E2 but was not dependent on the surface receptor CD81 (65). In another *in vitro* study, HCV viral particles were able to bind to recombinant human glycoprotein VI (GPVI) (69), which may be a potential receptor for HCV attachment on platelets. However, the role played by GPVI on HCV binding and entry in platelets has not been addressed (69). Endocytosed DENV-like particles have been also evidenced by ultrastructural analysis of platelets from patients with dengue (62). *In vitro* studies identified the mechanisms of DENV binding and internalization by platelets requiring DC-SIGN and heparan sulfate proteoglycans for viral attachment (58). When isolated platelets are infected with DENV *in vitro*, positive- and negative-sense viral RNA as well DENV non-structural protein 1 accumulate in platelets, indicating replication and translation of viral genome (58, 70). However, infective viral particles do not accumulate in platelet pellets or culture supernatants over time (58, 70). These data indicate that even though platelets support DENV replication, they do not assemble or release mature viral particles, strongly suggesting an abortive DENV infection in platelets.

We and others have shown that *in vitro* infection of platelets with DENV induces platelet activation (26, 64, 71, 72), which is inhibited by anti-DC-SIGN neutralizing antibodies (72). Beyond DENV binding to DC-SIGN, heparan sulfate or other surface receptors, DENV immunocomplexes formed with cross-reactive antibodies may enhance the infection of Fcγ receptor (FcγR)-bearing phagocytes in secondary DENV infections (73). Similarly, DENV capture by platelets is increased in the presence of antibodies (74), but whether this interaction leads to DENV replication and/or FcγR-mediated platelet activation remains unknown. Regarding this issue, immunocomplexes of influenza A (H1N1) virus with specific or cross-reactive (H3N2) antibodies activate platelets through FcγRIIA, increasing platelet degranulation and shedding of microparticles (MPs) (75). It remains unknown, however, whether virus attachment to platelet surface receptors is sufficient to initiate downstream activation of platelets or if virus internalization and replication are required for subsequent activation of intracellular PRR in platelets (Figure 1).

Among PRR involved in recognition and response against viruses, at least the endosomal TLR-3, -7, and -9 have already been demonstrated in megakaryocytes and platelets (23–25). Intracellular TLR-3 and -9 are highly expressed by megakaryocytes and there is evidence for at least TLR-3 functional responses in megakaryocytic cell lines (23, 25). Megakaryocytes infected with RNA viruses or stimulated with TLR-3 synthetic agonists poly I:C or poly A:U respond with increased type I IFN secretion and expression of ISGs (25, 29, 41). Similarly, isolated platelets stimulated with TLR-3 agonists translocate α-granule-stored factors (P-selectin and CD40L) to surface and have enhanced procoagulant responses to thrombin or other traditional agonists (25, 76). However, it is not clear if TLR-3 activation in platelets and megakaryocytes participates in immune response against viral infections.

While in nucleated cells the endosomal TLRs are restricted to intracellular compartment (77), TLR-3 and -9 in platelets have been shown to translocate to surface after platelet activation (8, 19, 23). In proplatelet-forming megakaryocytes, TLR-9 in electron-dense tubular system-related granules are transferred to platelet progeny. TLR-9-containing granules in platelets do not colocalize with α- or dense-granules, neither with TLR-7 or -8 or other endosomal proteins (23). Even though platelet TLR-9 is not expressed in endosomes, platelet stimulation promotes TLR-9 translocation to surface and pre-activated platelets are able to bind and respond to extracellular unmethylated CpG-containing oligodeoxynucleotide (ODN) (23). Besides ODN from viral or bacterial origin, TLR-9 also recognizes the endogenous DAMP carboxyalkylpyrrole (CAP) protein adducts formed during vascular oxidative stress. CAP adducts activate TLR-9–MyD88 signaling in platelets leading to platelet degranulation and aggregation *in vitro* and to *in vivo* thrombosis in atherosclerotic mice (78). However, whether and how surface-translocated TLR-9 in platelets are activated during viral infections deserves further investigation.

Toll-like receptor-7 signaling in platelets appears to be more similar to that of nucleated cells. During experimental encephalomyocarditis virus (ECMV) infection in mice, platelets are able to internalize and recognize viral RNA through TLR-7 (24). In this model, infected mice developed profound thrombocytopenia while platelets formed aggregates with neutrophils. Platelet TLR-7 also recognizes the synthetic agonist loxoribine which similarly causes platelet–neutrophil aggregates and thrombocytopenia in mice. Additional *in vitro* experiments with murine and human cells demonstrated that platelet–neutrophil aggregation in response to RNA virus or synthetic agonist require TLR-7 expression and endosomal-dependent signaling in platelets, but not in neutrophils. Importantly, transfusion of TLR-7-expressing platelets delayed the mortality of TLR-7-deficient mice after experimental ECMV infection, indicating that platelet TLR-7 may have important roles in immune response against RNA viruses (24).

Regardless of virus attachment to surface receptors or recognition by PRR in platelets, platelet activation can contribute to immune responses against viruses through a diversity of mechanisms including the release of chemokines that promote endothelial signaling and leukocyte migration or by physically interacting with leukocytes changing their responses (56, 79, 80). Recently, platelet factor 4 (PF4/CXCL4), a chemokine secreted exclusively by platelets and megakaryocytes, was identified as an essential mediator for neutrophil recruitment and virus clearance in influenza A infection (also see Platelet Activation in Influenza) (56). Another mechanism of protection involving platelet and neutrophils is platelet-mediated extrusion of neutrophil extracellular traps (NET). The ability of platelets to induce NET release has been extensively reported in bacterial infections, including in response to LPS (22, 81, 82). In an experimental model of myxoma virus infection, infected mice presented platelet–neutrophil aggregates and NET extrusion in the liver vasculature (79). In this model, platelet-mediated NET release protected liver cells from subsequent viral challenge (79). Platelets have been also identified as central players for preservation of spleen architecture and effective CD8^+^ T cell response against lymphocytic choriomeningitis arenavirus (LCMV), a murine model of viral hemorrhagic fever (80). In this model, partial platelet depletion to a degree that did not increase viral-induced hemorrhage significantly impaired viral clearance through defective assembly of virus-specific CD8^+^ T cell response (80). In a genetic model of hepatitis B virus infection, however, platelet-induced CD8^+^ T cell intrahepatic accumulation culminated in liver damage as consequence of cytotoxic activity of recruited lymphocytes toward elimination of infected cells (83). Even though the mechanisms of platelet activation and platelet–leukocyte signaling were not explored in these models, these data convincingly show that platelets play major roles in innate and adaptive immune responses against viruses.

## Platelet Activation in the Pathophysiology of Viral Infections

### Platelet Activation in HIV Infection

Because of combined antiretroviral therapy (ART), in the last decades the epidemiology of HIV has changed from high mortality by opportunistic infections in AIDS to long-term non-infectious complication of HIV infection (84, 85). However, even though sustained virologic control is achieved by ART, people living with HIV still experience increased mortality associated with higher rates of long-term comorbidities including cardiovascular diseases, depression, HIV-associated neurocognitive disorders, and non-AIDS cancers (86–90). Many of long-term complications in HIV-infected individuals are related to continuing immune suppression (87, 91, 92) and/or sustained inflammation (93–95). Ischemic thrombotic and cardiovascular events represent some of the most frequent long-term complications and leading cause of death among virologically suppressed HIV-infected subjects (86, 89, 91, 92, 96, 97). Considering the potential role for platelets in the cardiovascular risk of people living with HIV, much attention has been given on the implications of platelet activation in the pathogenesis of HIV infection.

Increased platelet activation in HIV-infected subjects and AIDS patients has been extensively reported in the last two decades (55, 98–104). In AIDS patients, markers of platelet activation correlate with patient viral loads and the nadir of CD4^+^ T cell counts (98–100, 102). P-selectin surface expression on platelets and plasma markers of platelet activation as soluble P-selectin and CD40L (sP-selectin and sCD40L) are increased in ART naïve patients and decrease during the first weeks after antiretroviral treatment (98, 102). However, there is evidence for sustained platelet activation after months to years of virologic suppression by ART (55, 103, 105, 106). Increased platelet activation in people living with HIV is associated with measures of inflammation and coagulopathy including elevated levels of TNF-α, tissue factor, and D-dimers (98, 99, 101). Consistently, antiplatelet therapy with aspirin has been shown to reduce not only platelet activation but also activation of monocytes and T cells from CD4^+^ and CD8^+^ subsets in HIV-infected subjects undergoing virologic suppression by ART (55).

Ultrastructural analysis of platelets from HIV-1-infected subjects or *in vitro*-infected platelets identify HIV-1 internalization in endosome-like vesicles (57, 107, 108). These ultrastructural studies show activation-associated morphology in HIV-containing platelets, indicating that platelets become activated after direct binding and internalization of HIV-1 viral particles (57, 108). In addition, HIV-1 transactivator of transcription (Tat), a viral protein that is released in the circulation of infected subjects, is able to activate platelets increasing the translocation and secretion of CD40L and P-selectin (109). Tat-mediated platelet activation occurs through its binding to β3 integrin and CCR3 chemokine receptor on platelets (109). Injection of recombinant Tat or Tat-expressing retrovirus increases platelet P-selectin expression and plasma levels of PF4 and sCD40L in mice (109–111). Nevertheless, other mechanisms beyond platelet interaction with HIV-1 or Tat may account for platelet activation in people living with HIV since platelet activation persists in virologically suppressed individuals (55, 103). One possible mechanism is platelet TLR-4 activation by LPS from microbial translocation, which is considered a central feature of HIV pathogenesis and has been documented to persist for years after initiation of ART (112). Other possibility is the effects of ART itself. Regarding this, abacavir-containing antiretroviral regimens have been associated with platelet hyperreactivity while raltegravir-based therapy was associated with reduced platelet activation (103, 113, 114). Ritonavir-containing protease inhibitor-based ART has been also associated with increased platelet activation when compared with samples obtained before ART initiation in a small cohort of protease-inhibitor naïve patients (104). In *in vitro* experiments, abacavir and its metabolite carbovir triphosphate have been shown to potentiate platelet responsiveness to prothrombotic agonists by competitively inhibiting the activity of guanylyl cyclase when compared with non-guanosine nucleotide analogs (115, 116). New studies are still necessary to investigate the impact of ART in platelet activation and long-term comorbidities in people living with HIV.

**Figure 2:**
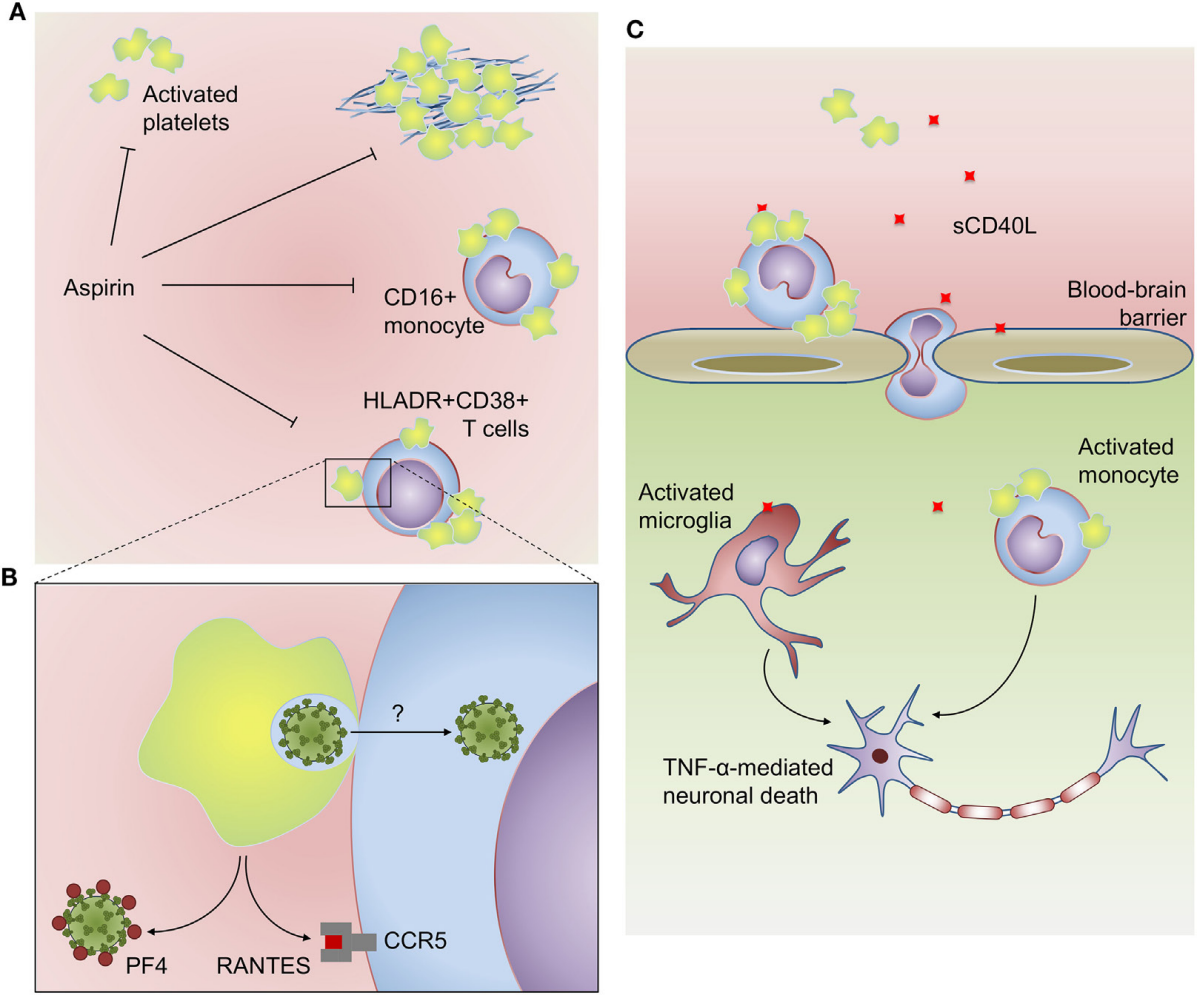
Platelets participate in inflammatory, virologic, and prothrombotic responses in HIV infection: **(A)** platelets from HIV-infected subjects have increased activation at baseline and hyperreactive aggregation under suboptimal prothrombotic stimuli. Activated platelets also form aggregates with CD16^+^ inflammatory monocytes and HLADR^+^CD38^+^ memory T cells in people living with HIV. HIV-infected subjects undertaken antiplatelet therapy with aspirin had reduced platelet activation, platelet hyperreactivity, and platelet–monocyte aggregates, as well as reduced activation of monocytes (sCD14) and T cells (HLADR^+^CD38^+^). **(B)** Platelets infected with high inoculum of reporter lentivirus are able to trans-infect susceptible T cells *in vitro*. Activated platelets can also inhibit human immunodeficiency virus 1 infection through the secretion of the HIV suppressive chemokines PF4/CXCL4 and RANTES/CCL5. **(C)** Platelet-derived CD40L plays a major role in HIV-associated neurocognitive disorders. Platelet-derived CD40L increases blood–brain barrier permeability and adhesion of GR1^+^CCR2^+^ leukocytes on brain microvasculature. CD40L-activated platelets induce monocyte migration though brain microvascular endothelial cells, and platelet–monocyte aggregates accumulate in brain microvasculature and parenchyma. Activated monocytes and microglia are more sensitive to CD40L-induced TNF-α secretion, which contributes to neuronal apoptosis. See the text for details and references.

Activated platelets in HIV-infected individuals express molecules including P-selectin, CD40L, and TF that participate in the thromboinflammatory state related to long-term comorbidities of HIV infection (98, 99, 103, 104, 235). In *ex vivo* aggregometry assay, platelets from HIV-infected subjects are more sensitive to aggregation under suboptimal prothrombotic stimulation (55, 113). In addition to prothrombotic responses, platelet activation participates in the immune and inflammatory network of HIV infection through diverse mechanisms involving secretion of stored factors and interactions with leukocytes as summarized in Figure 2 and discussed below.

Activated platelets interact with several classes of leukocytes including monocytes, neutrophils, and lymphocytes. These heterotypic aggregates are formed by the binding of platelet P-selectin to leukocyte P-selectin glycoprotein ligand 1 (PSGL-1), a molecular interaction that not only tethers the cells together but also signals gene expression and functional activating pathways in the leukocyte (1, 2, 117–119). Circulating platelet–monocyte and platelet–lymphocyte aggregates have been reported in increased numbers in HIV-infected subjects even after virologic suppression by ART (103, 120). Platelet aggregates formation with monocytes or with CD4^+^ and CD8^+^ T cells in people living with HIV correlate with P-selectin expression on platelets (120–122). In HIV-infected subjects under virologic suppression, aggregates of activated platelets with CD4^+^ and CD8^+^ T cells form preferentially with lymphocytes of memory phenotype (120). Interestingly, memory lymphocytes isolated from HIV-infected individuals have increased avidity for recombinant P-selectin *ex vivo*, suggesting that besides platelet activation, T cell activation state also contributes to platelet–lymphocyte adhesion in HIV infection (120). Platelet–monocyte aggregates in HIV-infected people are more frequent among CD16^+^ inflammatory monocytes and associates to increased levels of sCD163, a marker of monocyte activation (121, 122). While, similarly to lymphocytes, inflammatory monocytes may be more avid targets for activated platelets, it is tempting to speculate whether platelet adhesion contributes to monocyte or lymphocyte inflammatory activation in HIV infection. However, new studies are still necessary to determine the effects of platelet–leukocyte aggregates on monocyte- and T cell-mediated responses during HIV infection.

Among stored factors secreted upon platelet activation, two major chemokines from α-granules, RANTES/CCL5 and PF4/CXCL4, are considered endogenous inhibitors of HIV-1 replication regarding their ability to bind HIV-1 coreceptor CCR5 and HIV-1 GP120, respectively (123, 124). Together with other chemokine ligands of HIV-1 coreceptors MIP-1α/CCL3 and MIP-1β/CCL4 for CCR5 and SDF-1/CXCL12 for CXCR4 (123), platelet-derived RANTES and PF4 may be central mediators in the control of HIV-1 replication *in vivo* [for more details on HIV-1 suppressive chemokines see Ref. (125)]. Accordingly, genetic polymorphisms involved with the expression of these chemokines and their receptors have been reported as key determinants of the outcome in HIV-1 infection (126–129). These epidemiologic studies are supported by ultrastructural analysis of platelets from HIV-1-infected subjects or *in vitro*-infected platelets that show evidence for α-granules fusion with virus-containing compartments indicating HIV inactivation and/or degradation by α-granule proteins (57, 108). In addition, coculture with platelets is able to prevent T cell infection through PF4-dependent mechanisms regardless of CCR5 or CXCR4 coreceptor tropism (61, 124). In another study, however, when platelets were infected with HIV-1 in a high viral load and exposed to T cells few hours postinfection, platelets were able to trans-infect T cells instead of preventing their infection (14). These results suggest that platelets may have a dual role in HIV-1 spreading, being able to inactivate endocytosed viruses or shelter them depending on viral load and platelets α-granule content (14, 61) (Figures 1 and 2B). Of note, platelet exhaustion of α-granule chemokines has been reported in association with high viral load in HIV/AIDS patients (98).

Other platelet-derived mediators as TGF-β and CD40L have been implicated in pathologic mechanisms of HIV-associated long-term comorbidities as consequence of infection or ART (111, 130–132). TGF-β from activated platelets has been identified as a critical factor for heart tissue fibrosis and cardiac dysfunction in mice chronically treated with the antiretroviral ritonavir (132); and CD40L, besides being a key mediator for B cell germinative center formation and isotype switching, plays immunopathogenic roles in HIV-associated neurocognitive disorders (Figure 2C) as discussed below (109, 111, 121).

The levels of sCD40L are increased in plasma and cerebrospinal fluid from patients with HIV-associated cognitive impairment compared with HIV-infected subjects without cognitive damage (130). In mice experimentally infected with transgenic viruses (retrovirus expressing HIV Tat or HIV expressing murine leukemia virus GP80 in replacement of GP120) and in Tat-injected mice, platelet-derived CD40L is a crucial component for increased blood–brain barrier permeability and leukocyte recruitment to brain microvasculature (109, 111, 131). CD40L reciprocally increases platelet activation promoting the formation of platelet–monocyte aggregates (121). Adhesion of CD40L-activated platelets to monocytes increase their ability to transmigrate brain microvascular endothelial cells *in vitro*; and increased numbers of platelet–monocyte aggregates have been observed on microvascular bed and in vascular lumen of brain sections from patients who died by HIV-associated encephalopathy (121). Recruited monocytes and activated resident microglia secrete pro-inflammatory cytokines in response to CD40L, including TNF-α, amplifying local inflammation, and neuronal death, as suggested by *in vitro* experiments with Tat-activated monocytes and microglia (130). These evidences from infected patients together with *in vivo* and *in vitro* studies suggest that platelet-derived CD40L plays a central role in HIV-associated neurocognitive disorder.

### Platelet Activation in the Pathogenesis of Dengue

Dengue is an arthropod-born viral disease caused by one of four antigenically related DENV serotypes (DENV-1 to -4). It is the most frequent hemorrhagic viral disease and re-emergent infection in the world (133, 134). Recently, it was estimated that over 2.5 billion people live in high-risk transmission areas with more than 90 million symptomatic infections occurring annually (134). DENV infection induces a spectrum of clinical manifestations that range from mild self-limited dengue fever to life-threatening severe dengue. While mild dengue presents as undifferentiated febrile illness, severe dengue syndrome progress with hemodynamic dysfunction including coagulopathy and vasculopathy associated with hypovolemia, hypotension, shock, organ dysfunction, and eventually death (133, 135–137). Thrombocytopenia is a common feature in dengue syndromes, and the drop of platelet counts is temporally coincident with the hemodynamic instability and progression to severity, while its recovery associates with clinical improvement and hospital discharge (136, 138–142). The pathophysiologic mechanisms underlying severe dengue cases are not completely understood. Overwhelming immune activation with increased levels of cytokines and other pro-inflammatory mediators that target the vascular endothelium is considered to favor DENV pathology and severity (140, 143–146). Activation of various immune cells including B and T cells, monocytes, macrophages, and dendritic cells has been shown to participate in this process (147–152). Even through thrombocytopenia is a hallmark of dengue infection, the role played by platelets in dengue immunopathology was only recently addressed.

**Figure 3:**
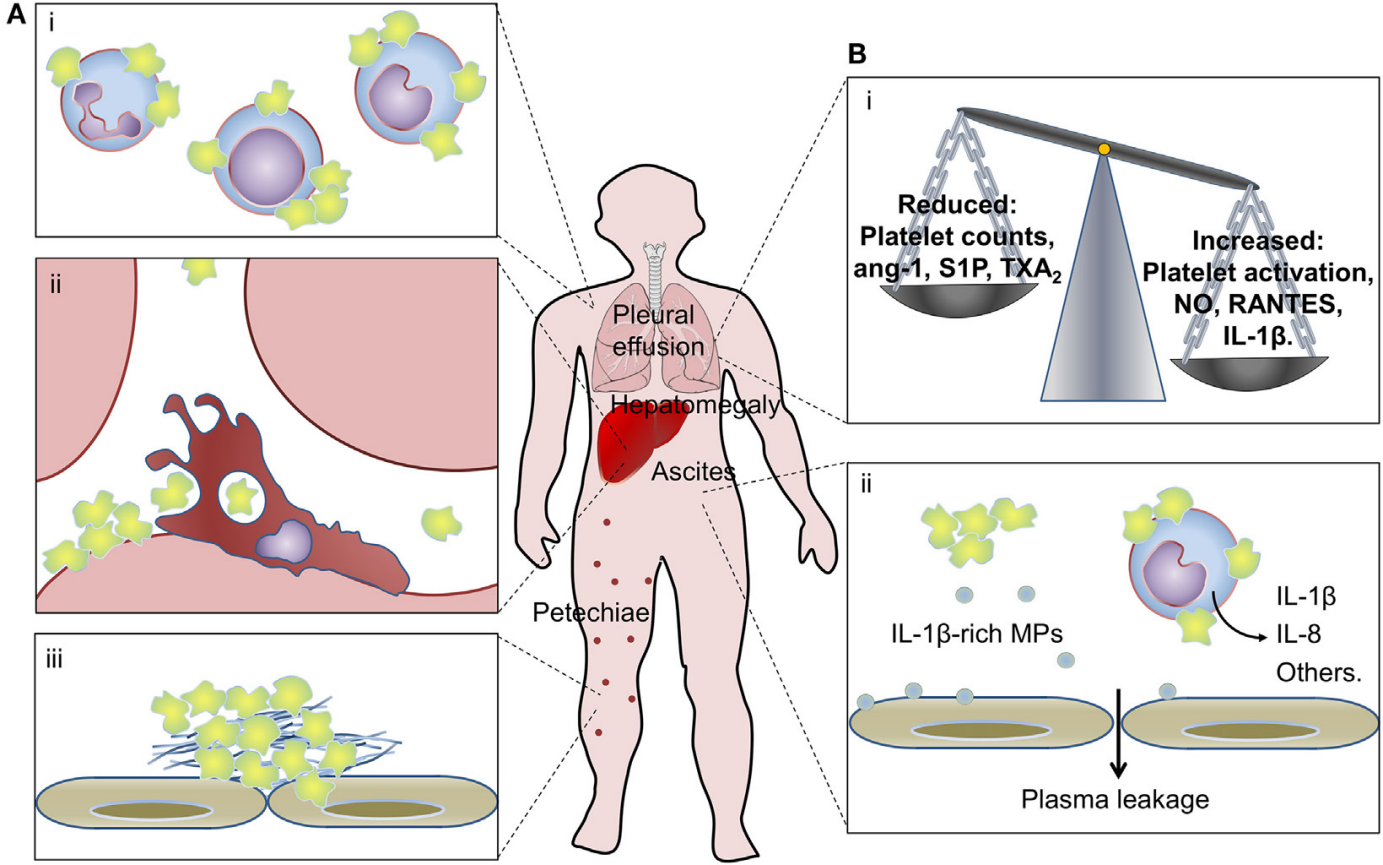
Platelets in dengue pathogenesis. **(A)** Peripheral mechanisms of thrombocytopenia in dengue: (i) platelet–leukocyte aggregates are formed with monocytes, lymphocytes, and neutrophils in the circulation of dengue patients; (ii) increased platelet sequestration in the liver of patients with dengue hemorrhagic fever/dengue shock syndrome and clearance of apoptotic platelets by resident macrophages through phosphatidylserine-mediated phagocytosis; and (iii) platelet adhesion and platelet clot formation on microvascular bed at the periphery may all contribute to thrombocytopenia in dengue. **(B)** Contributions of platelet activation and thrombocytopenia to inflammatory amplification and increased vascular permeability in dengue: (i) thrombocytopenia and reduced levels of platelet-derived endothelium-stabilizing factors alongside increased production of pro-inflammatory vasoactive factors by activated platelets that remain in circulation may contribute to dengue-associated vasculopathy; (ii) platelet synthesis of IL-1β and release of IL-1β-containing microparticles (MPs) associate with increased vascular permeability in dengue patients, and IL-1-β-rich MPs shed from dengue virus-infected platelets increase endothelial permeability *in vitro*; activated platelets from dengue-infected patients also induce pro-inflammatory cytokine secretion by monocytes. See the text for details and references.

Platelet activation has been demonstrated in patients with dengue and increased platelet activation associated with disease severity (26, 54, 64, 72, 153, 154). Surface markers of activation including P-selectin and CD63 expression (translocated from α- and dense-granules, respectively), phosphatidylserine exposure, and inside-out activation of α_IIb_β_3_ integrin all correlate with the decline of platelet counts during dengue infection (64, 72, 155). Increased platelet activation may contribute to platelet loss by mediating platelet deposition in the microvascular bed at the periphery. In agreement, platelet aggregates and fibrinogen deposition have been detected in microvessels of postmortem histology and skin biopsies from severe dengue cases (156, 157). In a flow model, perfusion of DENV-infected whole blood on histamine-activated endothelial cells formed increased area of adhered platelets and platelet-von Willebrand factor strings compared with uninfected blood (64). Platelets have been also shown to adhere on DENV-infected endothelial cells *in vitro*, which reciprocally increased platelet activation (158). Aggregation of activated platelets with leukocytes may also contribute to thrombocytopenia in dengue, and circulating platelet–leukocyte aggregates have been shown among monocytes, lymphocytes, and granulocytes from dengue patients (54, 159). Accordingly, the levels of platelet–monocyte aggregates in the circulation of dengue patients negatively correlate with platelet counts (54). The participation of platelet–leukocyte aggregates (as well other platelet-mediated responses) in inflammatory amplification and dengue pathogenesis will be discussed further in this section and is highlighted in Figure 3.

The exact triggers of platelet activation in dengue infection are not completely clear. As discussed earlier, the mechanisms of DENV binding and replication in platelets and those involved in activation of DENV-infected platelets have been investigated (58, 64, 72). Consistently, DENV genome copies in platelets positively correlate with platelet activation in acute dengue infection (64). However, platelet activation in patients with dengue is maximal at the critical phase of infection, when DENV particles are no longer circulating (72, 153), indicating the existence of additional mechanisms governing platelet activation in non-viremic phases of infection. Mechanisms possibly involved in platelet activation in dengue include platelet adhesion to activated endothelium (158), increased thrombin generation (138, 160–162), and increased production of pro-inflammatory mediators as platelet-activating factor (PAF) (163–165). Regarding the latter, experimental DENV infection in mice lacking PAF receptor results in mild disease with reduced thrombocytopenia, inflammation, and mortality (166). Recently, a quantitative proteome approach of platelets from patients with dengue revealed novel mechanisms and pathways of platelet activation involving the binding of cell-free histone H2A (154). Cell-free histones are recognized DAMP signals that activate platelets through TLR-2 and -4 *in vitro* and *in vivo* (167–169). Injection of histones in mice leads to histone accumulation in sites of thrombosis and to thrombocytopenia (168–170). In dengue patients, increased levels of circulating cell-free histone H2A and histone sequestration by circulating platelets associate with disease severity (154). When platelets from healthy volunteers are exposed to plasma from dengue patients, cell-free histone H2A binds to and activates platelets *ex vivo*, indicating that circulating cell-free histones may contribute to platelet activation in dengue (154).

Aside from platelet activation and intravascular thrombosis, increased platelet apoptosis may contribute to thrombocytopenia in dengue infection. Increased rates of apoptotic platelets circulate in dengue-infected patients (54, 72, 155). *In vitro* infection with any serotype of DENV also culminates in the activation of an intrinsic cell death program in platelets that involves mitochondrial dysfunction and activation of apoptotic caspases (64, 72, 155). Patients with dengue hemorrhagic fever (DHF) present higher frequencies of platelet apoptosis compared with mild dengue (155). Accordingly, platelet life-span is reduced in DHF, and patients with DHF/DSS present increased platelet sequestration in the liver (171). In parallel *ex vivo* experiments, cultured macrophages phagocytized apoptotic platelets from dengue patients chiefly depending on phosphatidylserine recognition by the phagocyte (155). These studies suggest that platelet retention in the reticuloendothelial system with phagocytosis of apoptotic platelets by resident macrophages may represent a key mechanism of thrombocytopenia in dengue.

Besides platelet apoptosis and aggregation at the periphery, central mechanisms of bone marrow suppression may also contribute to thrombocytopenia in dengue (172, 173). DENV propagation in bone marrow (33, 174, 175) may contribute to suppression of hematopoiesis in all lineages, including the megakaryocytic (174, 176, 177). Bone marrow aspirates from dengue patients show moderate hypocellularity and arrest of maturation in granulocyte, megakaryocyte, and erythroid/myeloid precursors (178–181). In agreement, experimental DENV infection of humanized mice shows transient thrombocytopenia with reduced numbers of human megakaryocytic progenitors and megakaryocytes in the marrow (182). In *in vitro* experiments, DENV infection of megakaryocyte progenitor cells culminates in loss of their proliferative capacity and cell death by apoptosis (177). Regarding mature megakaryocytes, it has been demonstrated in non-human primates and *ex vivo* infection of human marrow cells that megakaryocytes are the main target for DENV in the marrow (32, 33), but whether DENV infection of mature megakaryocytes impacts platelet production, quantitative or qualitatively, remains to be explored.

Thrombocytopenia may be involved in the increased vascular permeability of dengue patients. Platelets participate in the regulation of endothelial barrier function and are required for the maintenance of basal endothelial permeability in physiological conditions (2, 183, 184). In *in vitro* experiments, isolated platelets or supernatants from rested platelets are able to reduce the permeability of endothelial monolayers in a concentration-dependent fashion (185–188). Platelet-rich plasma is also able to revert TNF-α-induced endothelial permeability *in vitro* and LPS-induced alveolar-capillary leak in experimental endotoxemia in mice (189). Various platelet-derived proteins and lipid mediators have been identified as vascular endothelium-stabilizing factors including lysophosphatidic acid, sphingosine-1-phosphate (S1P), thromboxane A_2_ (TXA_2_), and angiopoietin-1 (188–193). In agreement, reduced plasma levels of angiopoietin-1 alongside increased levels of its antagonist angiopoietin-2 are associated with shock presentation in children with DHF (194). Similarly, plasma levels of TXA_2_ and S1P are reduced in patients presenting ultrasound-evidence of plasma leakage or that progress to shock (195–197). These results together with the association between thrombocytopenia and plasma leakage in dengue patients (136, 138–142) suggest that platelets at steady state are required for preservation of vascular barrier integrity, and reductions in platelet counts and platelet-derived endothelium-stabilizing factors might increase vascular permeability in dengue. Paradoxically, activated platelets participate in inflammatory processes that trigger increased vascular permeability as well.

Activated platelets in patients with dengue or platelets infected with DENV *in vitro* secrete various factors with vasorelaxing or endothelial activating functions, including small molecules (serotonin and nitric oxide) (153, 198, 199), granule-stored chemokine (RANTES/CCL5 and PF4/CXCL4) (154), and newly-synthesized IL-1β (26). Among these factors, at least nitric oxide, RANTES, and IL-1β have been linked to disease severity and vascular instability in dengue-infected patients (140, 200–204). Similarly, platelet activation markers have been associated with measures of plasma leakage in patients with dengue (26, 54, 153). We recently reported increased expression of IL-1β in platelets from patients with dengue and in DENV-infected platelets *in vitro*. Newly synthesized pro-IL-1β was processed by inflammasome-dependent caspase-1 activity, as demonstrated in platelets from infected patients and in functional assays using inflammasome and caspase-1 inhibitors *in vitro* (26). In this model, IL-1β processing and secretion required the generation of reactive oxygen species in mitochondria as an endogenous signal for NLRP3 activation (Figure 1). Biologically active mature IL-1β was released from platelets in suspension and in IL-1β-rich MPs. In patients with dengue, IL-1β expression in platelets and platelet-derived MPs and caspase-1 activity in platelets correlated with clinical signs of increased vascular permeability; and MPs recovered from DENV-infected platelets increased endothelial cell permeability *in vitro*, which was blocked by IL-1 receptor antagonist. These observations in platelets from patients with dengue and from *in vitro* infection models and functional assays provided evidence for NLRP3 inflammasome activation in platelets culminating in the release of IL-1β-containing MPs as an important pathogenic mechanism for vasculopathy in dengue syndrome (26).

Platelet–leukocyte aggregates also participate in leukocyte immunoregulation and inflammatory response in dengue. As mentioned before, platelets form aggregates with lymphocytes, monocytes, and granulocytes during dengue infection (54, 159). Circulating platelet–monocyte and platelet–neutrophil aggregates were also demonstrated in a model of dengue-induced hemorrhage in rhesus macaques (205). In this model, microscopic evidence of platelet–monocyte aggregates and platelet phagocytosis by monocytes in peripheral blood was also provided (205). In recent experiments from our group, *ex vivo* heterologous interactions of platelets from dengue-infected patients with monocytes from healthy volunteers demonstrated the ability of activated and apoptotic platelets from infected patients to modulate the synthesis and secretion of IL-1β, IL-8, and IL-10 by monocytes. The same responses were not achieved by platelets from healthy volunteers interacted with monocytes from heterologous healthy subjects or dengue patients (54). Complementary *in vitro* experiments demonstrated that modulation of cytokine secretion by monocytes required P-selectin-mediated adhesion and recognition of phosphatidylserine on apoptotic platelets, which provided a previously unrecognized signal for IL-10 secretion in platelet–monocyte complexes (54). Beyond cytokines, activated platelets have been shown to trigger the synthesis of chemokines, adhesion molecules, metalloproteinase-9, and cyclooxygenase-2 by monocytes (118, 119, 206, 207), which were all implicated in dengue immunopathology in other studies (195, 208–213).

The consequences of platelet–neutrophil and platelet–lymphocyte interactions in dengue pathogenesis is far less explored. Regarding platelet interaction with lymphocytes, the ability of platelets to cross-present exogenous protein antigens to CD8^+^ T cells through major histocompatibility complex (MHC) class I was recently demonstrated *in vitro* and in experimental cerebral malaria *in vivo* (214). In this study, platelets effectively activated antigen-specific CD8^+^ T cells through presentation of pathogen-derived antigen in MHC class I (214). Very recent experiments investigating the ability of mature megakaryocytes to process exogenous proteins and present their peptides in MHC class I indicate that megakaryocytes also trigger CD8^+^ T cell activation and proliferation *in vitro* and *in vivo*. More importantly, megakaryocytes are able to transfer antigen-loaded MHC class I complexes to platelets during thrombopoiesis (31). Platelets isolated from patients with dengue have increased expression of MHC class I as evidenced by mass spectrometry-based proteome and western blot (154). This increased MHC class I content may derive from megakaryocytes, which have been identified as the main target cells for DENV infection in marrow (32, 33); or from DENV infection of platelets at circulation (62–64). In *in vitro* experiments, platelets infected with DENV increased MHC class I expression and surface display through mechanisms depending on proteasome activity (154). Whether peptides processed by platelet proteasome/immunoproteasome and presented in MHC class I on platelets derived from viral antigens or self-proteins requires further investigation. In experimental models or patients with idiopathic thrombocytopenic purpura and in platelet transfusion-refractory individuals, platelet MHC class I-mediated CD8^+^ T cell cytotoxicity leads to platelet clearance and inflammatory cytokine secretion (31, 214–219), both important pathogenic mechanisms in dengue. Nevertheless, new studies are still necessary to investigate the role played by platelet MHC class I expression and platelet–lymphocyte interactions in dengue pathogenesis, specially thrombocytopenia and cytokine storm.

### Platelet Activation in Influenza

**Figure 4:**
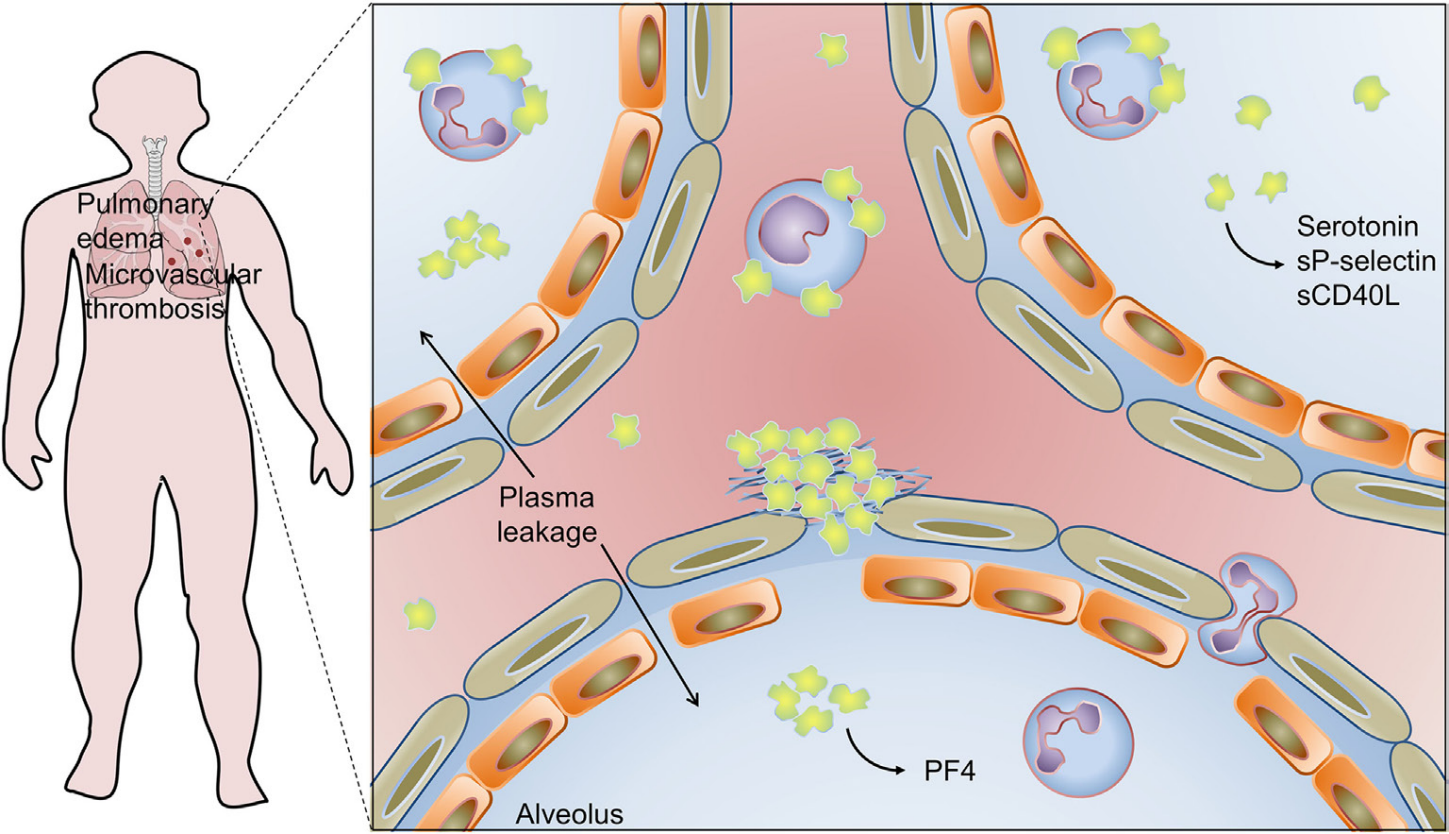
Platelet activation and infiltration to airways in influenza pneumonia: pulmonary edema and microvascular thrombosis with platelet–fibrin clot deposition in areas of disrupted alveolar-capillary barrier integrity are histopathological features of influenza pneumonia. Platelets, platelet–platelet aggregates, and platelet–neutrophil aggregates have been also detected in extravascular space in the lungs, and platelet–monocyte aggregates have been demonstrated in circulation. Activated platelets secrete serotonin, sCD40L, sP-selectin, and PF4 in the airways. Platelet activation is involved in pathophysiologic mechanisms of lung injury and pulmonary edema in influenza. On the other hand, platelet secretion of PF4 is an essential mechanism for neutrophil recruitment to the lungs and innate immunity against influenza virus. See the text for details and references.

Influenza pneumonia, caused by virulent strains of influenza A virus (IAV), remains a major global health problem with seasonal influenza epidemics and unexpected pandemics that have occurred for more than a century. For example, the virulent H1N1 strain of influenza identified in the 2009 flu pandemic caused increased morbidity and mortality worldwide (220–222). Patients admitted in intensive care unit (ICU) with severe influenza pneumonia usually presented acute lung injury and acute respiratory distress syndrome (ALI/ARDS) which have exacerbated immune response and increased systemic and airway inflammation as central features of pathogenesis (221, 223, 224). Pathophysiologic mechanisms of severe influenza pneumonia include overwhelming immune activation at the airways resulting in alveolar-capillary barrier damage, edema, pulmonary microvascular thrombosis, and progressive loss of respiratory capacity (224–226). Because of disrupted alveolar-capillary barrier integrity in influenza pneumonia, platelets may interact with influenza virus or influenza-IgG immunocomplexes even if the virus is restricted to alveoli and airways compartment (75, 227). In addition, there has been evidence for platelet activation by locally generated agonists and damage signals (227, 228). The participation of platelets in local immune and inflammatory responses during influenza infection (Figure 4) makes a perfect example of platelet activities at the extravascular space.

Increased platelet activation and increased formation of platelet–monocyte aggregates have been reported in the blood of critically ill H1N1 influenza patients presenting ALI/ARDS (229). In this study, platelet activation and platelet–monocyte aggregates’ formation were higher in H1N1 influenza patients compared with patients with bacterial pneumonia at ICU (229). Activated platelets and platelet–monocyte aggregates have been also shown in influenza vaccinated subjects (230–232). Increased platelet activation and platelet–monocyte aggregation after influenza vaccination correlated with the expansion of inflammatory CD14^high^CD16^+^ and reduction of classical CD14^high^CD16^−^ monocyte subsets, which was prevented by antiplatelet therapy with aspirin or clopidogrel (230, 232). Complementary *in vitro* experiments indicated that concurrent signaling from P-selectin-PSGL-1 binding and secreted prostaglandin E_2_ expanded the subset of CD16^+^ inflammatory monocytes in platelet–monocyte cocultures (230). However, whether and how platelet–monocyte aggregates contribute to the expansion of inflammatory monocytes in influenza or other infections in humans remains to be demonstrated.

Histopathological studies of lungs in experimental influenza A infection in mice and autopsies from patients who expired from H1N1 influenza documented frequent microvascular thrombosis in areas of disrupted integrity of alveolar-capillary barrier (56, 226–228). In influenza A-infected mice, platelet infiltration to lung parenchyma was evidenced by CD41 and PF4 immunohistochemistry, transmission electron microscopy, and platelet counting in bronchoalveolar lavage (BAL) (56, 75, 227, 228). Ultrastructural analysis of infiltrated platelets shows evidence of platelet activation and formation of platelet–neutrophil aggregates, which are in agreement with increased levels of serotonin, sP-selectin, and sCD40L in BAL (227, 228). In these models, platelet activation has been observed in both airways and blood from infected mice, advocating in favor of multiple (parallel or sequential) mechanisms for platelet activation (56, 227, 228). These mechanisms may involve, in addition to direct contact with the virus or virus-IgG immunocomplexes, the generation of agonists and damage signals that activate infiltrated and circulating platelets in severe influenza pneumonia (75, 227, 228). Recent *in vitro* experiments investigating platelet responses to H1N1 in the presence of influenza immune serum demonstrated that complete thromboinflammatory phenotype of activated platelets depends on the synergistic activation of FcγRIIA by immunocomplexes and protease activating receptor (PAR) by thrombin (75).

Studies of experimental influenza A infection plus pharmacological and genetic models have supported the participation of platelets in the pathogenesis of airway inflammation and lung injury. Influenza A infection together with PAR-4 agonist or antagonist were able to, respectively, exacerbate or recover lung inflammation, plasma leakage and mortality with no change on influenza viral load (227). Genetic deficiency of integrin α_IIb_β_3_ or treatment with the integrin α_IIb_β_3_ inhibitor eptifibatide reduced influenza- or influenza plus PAR-4 agonist-induced platelet infiltration and activation in the lungs, and rescued infected mice from lung injury and mortality (227). Other antiplatelet drugs including aspirin and P2Y purinergic receptor inhibitors also protected influenza-infected mice from platelet accumulation in lungs and mortality (227, 233). In complementary *in vitro* experiments, it was demonstrated that platelet attachment to infected pulmonary microvascular endothelial cells depended on integrin-mediated adhesion and was also blocked by pharmacological inhibitors of platelet activation (233). Very recent studies investigating the role of extracellular histones in influenza-mediated lung injury and vascular thrombosis has evidenced the deposition of cell-free histones in association with activated platelets and neutrophils in areas of alveolar damage in lungs of infected mice (228). Increased levels of cell-free histones have been observed in BAL from influenza-infected mice and in nasal wash from influenza A, but not influenza B, infected patients (228, 234). Even though this study has demonstrated activated platelets in BAL of infected mice, the particular role of cell-free histones in platelet activation and platelet infiltration in the airways has not been addressed (228). Altogether, these experiments indicate that pathogenic mechanisms involving activation-dependent platelet adhesion, infiltration, and inflammatory response in the lungs contribute to influenza pneumonia and ARDS. These evidences from experimental infection models shed light on novel targets for therapeutic intervention in influenza-associated ALI/ARDS. Accordingly, supplementation of antiviral therapy with antiplatelet drugs or antihistone antibodies has improved histological index of lung injury and increased the survival of infected mice when compared with antiviral therapy alone (228, 233).

Another recent work indicates that platelets have protective activities in innate immune response against IAV. Increased levels of PF4 accumulate in blood and lung of mice experimentally infected with H1N1 influenza. Based on genetic models, PF4 in blood and lungs from infected mice have essential roles in innate mechanisms of viral clearance and survival. Experimental infection of PF4-deficient mice resulted in exacerbated lung inflammation, alveolar damage, and increased mortality (56). Mice lacking PF4 were unable to provide effective clearance of the virus even though specific T and B cell-mediated adaptive immunity were responsive (56). Deficient viral clearance was related to insufficient production of neutrophil chemoattractant chemokines and, consequently, to lower neutrophil migration to the circulation and lungs. PF4 intravenous injection or instillation in the lungs recovered the levels of chemokines, infiltration of neutrophils, and survival of PF4-deficient mice (56). These experimental observations together with the ones aforementioned indicate that platelets accumulate at intra- and extravascular space in the lungs during influenza A infection, and that they are effectors of both host protection and, in severe influenza pneumonia, lung injury (56, 227).

## Conclusion

Emerging evidences identify platelets as dynamic cells that participate in inflammation and prothrombotic responses in many vascular and inflammatory processes, including viral infections. New platelet functions have emerged over time and platelets are getting increasingly recognized as immune cells. Pathophysiological mechanisms involving platelet responses have been reported in HIV infection, dengue fever, and influenza pneumonia in naturally infected patients and in experimental infection models as discussed here. Thrombocytopenia, platelet secretion of stored and newly synthesized factors, and complex interactions with leukocytes comprise platelet features that may have both injurious and protective immune consequences in viral infections. Increasing our understanding on immunoregulatory functions of platelets in viral infections will undoubtedly improve our knowledge on diseases pathogenesis, clinical management, and therapeutic options.

## Author Contributions

EH, FB and PB contributed to development of the concepts and design of the review article. EH wrote the manuscript and prepared the figures. PB and FB reviewed the manuscript.

## Conflict of Interest Statement

The authors declare that the research was conducted in the absence of any commercial or financial relationships that could be construed as a potential conflict of interest.
